# *Leishmania donovani* Argininosuccinate Synthase Is an Active Enzyme Associated with Parasite Pathogenesis

**DOI:** 10.1371/journal.pntd.0001849

**Published:** 2012-10-18

**Authors:** Ines Lakhal-Naouar, Armando Jardim, Rona Strasser, Shen Luo, Yukiko Kozakai, Hira L. Nakhasi, Robert C. Duncan

**Affiliations:** 1 Laboratory of Emerging Pathogens, Division of Emerging and Transfusion Transmitted Diseases, Center for Biologics Evaluation and Research (CBER), Food and Drug Administration (FDA), Bethesda, Maryland, United States of America; 2 Institute of Parasitology, McGill University and the Centre for Host-Parasite Interactions, Quebec, Canada; 3 Laboratory of Chemistry, Division of Therapeutic Proteins, Center for Drug Evaluation and Research (CDER), Food and Drug Administration (FDA), Bethesda, Maryland, United States of America; Louisiana State University, United States of America

## Abstract

**Background:**

Gene expression analysis in *Leishmania donovani* (Ld) identified an orthologue of the urea cycle enzyme, argininosuccinate synthase (LdASS), that was more abundantly expressed in amastigotes than in promastigotes. In order to characterize in detail this newly identified protein in *Leishmania*, we determined its enzymatic activity, subcellular localization in the parasite and affect on virulence *in vivo*.

**Methodology/Principal Findings:**

Two parasite cell lines either over expressing wild type LdASS or a mutant form (G128S) associated with severe cases of citrullinemia in humans were developed. In addition we also produced bacterially expressed recombinant forms of the same proteins. Our results demonstrated that LdASS has argininosuccinate synthase enzymatic activity that is abolished using an ASS specific inhibitor (MDLA: methyl-D-L-Aspartic acid). However, the mutant form of the protein is inactive. We demonstrate that though LdASS has a glycosomal targeting signal that binds the targeting apparatus *in vitro*, only a small proportion of the total cellular ASS is localized in a vesicle, as indicated by protection from protease digestion of the crude organelle fraction. The majority of LdASS was found to be in the cytosolic fraction that may include large cytosolic complexes as indicated by the punctate distribution in IFA. Surprisingly, comparison to known glycosomal proteins by IFA revealed that LdASS was located in a structure different from the known glycosomal vesicles. Significantly, parasites expressing a mutant form of LdASS associated with a loss of *in vitro* activity had reduced virulence *in vivo* in BALB/c mice as demonstrated by a significant reduction in the parasite load in spleen and liver.

**Conclusion/Significance:**

Our study suggests that LdASS is an active enzyme, with unique localization and essential for parasite survival and growth in the mammalian host. Based on these observations LdASS could be further explored as a potential drug target.

## Author Summary

Leishmaniasis is a neglected tropical disease that continues to pose a public health threat worldwide due to the absence of an effective vaccine, drug toxicity and parasite resistance. In an attempt to identify new potential drug targets, we focused our research on *Leishmania donovani* argininosuccinate synthase (LdASS), which is more highly expressed in the virulent form of the parasite. Using two cell lines that over expressed the wild type or a mutant form of LdASS, we demonstrated that LdASS has argininosuccinate synthase activity, which is absent in the mutant form containing the G128S point mutation. Infection of mice with the cell line over expressing a mutant LdASS had a negative dominant effect as indicated by the reduction in parasite load. LdASS is localized to large cytosolic complexes and a small portion is in a new vesicular subset different from the known glycosomes. Thus LdASS constitutes a new virulence factor that may be a potential drug target.

## Introduction

Leishmaniasis represents a group of parasitic diseases caused by infection with a parasite of the genus *Leishmania* that is transmitted to the host by the phlebotomine sandfly bite. *Leishmania* species have a digenetic life cycle alternating between two forms: the extracellular promastigote form that resides in the sandfly gut and the aflagellated intracellular amastigote form present in the phagolysosome of host macrophages [1]. Understanding the host-parasite interaction at the molecular level allows the identification of molecules involved in pathogenesis that may be targeted to control the infectious disease. Studies conducted in our laboratory have identified such virulence associated proteins [2]–[3].

Additional uncharacterized proteins have been identified in studies of *L. donovani* gene expression. Argininosuccinate synthase (ASS) was identified as a biomarker of attenuation in the *L. donovani* centrin deleted cell line [4]. In that cell line, ASS expression was lost when parasites differentiate into the amastigote stage and the attenuated phenotype is manifest. Further, it was shown that the virulent strain expresses a higher level of ASS in the amastigote life cycle stage responsible for the disease [4]. In the current study, functional analysis of LdASS is being demonstrated for the first time. Thus ASS characterization in *Leishmania* could reveal whether it is a virulence factor for drug therapy targeting or for manipulation to create a genetically defined attenuated parasite vaccine candidate.

ASS is a key enzyme of the urea cycle that catalyses the rate-limiting step in the conversion of L-citrulline to L-arginine. ASS has been recognized as one of the key factors regulating L-arginine metabolism [5]. ASS was also found to have a rate-limiting role in high output nitric oxide (NO) synthesis [6]. ASS has been purified from many species [7] and was extensively studied in humans and bacteria. The ASS gene is well conserved between human, bacteria, bovine, rat, yeast and mouse [8]. In Kinetoplastids, the ASS gene is found in *Leishmania* but not in *Trypanosoma* (*T. brucei* or *T. cruzi*) genomes and seems to have been acquired by lateral gene transfer from bacteria [9]. The X-ray crystal structures of ASS from bacteria (*E. coli*) [7], [10], *Thermus thermophilus* [11]–[12] and humans [13] have been solved and showed that ASS is arranged in three domains: a nucleotide-binding domain containing an “N-type” ATP pyrophosphatase consensus sequence, the synthetase domain, and a C-terminal oligomerization domain [13]. All ASS molecules studied so far are homotetramers with a subunit size of about 46 kDa [10], [14]. The oligomeric arrangement of ASS as a tetramer is conserved in the bacteria and human structures [13]. It was clearly demonstrated that ATP binding to the ASS molecule results in a large rigid body conformational change shifting the nucleotide binding domain toward the synthetase domain as an essential part of is the enzymatic mechanism [7].

The subcellular localization of ASS varies depending on the tissue [15] and the regulation of its expression is cell/tissue specific [8]. Bioinformatic analysis of the *L. major* coding sequence predicted ASS to be located in the glycosome [16]. Glycosomes are membrane bound organelles found only in the kinetoplastid protozoa [17]–[18] that compartmentalize several important metabolic pathways including glycolysis, purine salvage and pyrimidine biosynthesis, that are essential for parasite survival [19]. Glycosomes in *Leishmania* and peroxisomes in human, yeast and plants have a common evolutionary origin [20]. Based on their isopycnic point, purification of glycosomes over a sucrose gradient was demonstrated in *Leishmania* [21]. The targeting to the glycosomes is mediated either by a C-terminal type 1 peroxisomal targeting signal (PTS1), variant of the SKL tripeptide [22] or a more degenerate PTS2 signal, which is typically located proximal to the N-terminus and has the consensus motif R/K-L/V/I-X5-Q/H-L/A [23].

In humans, metabolic disturbances associated with impairment in ASS gene function result in citrullinemia type I [24]. Among the associated mutations in conserved amino acids, a mutation of glycine to serine at position 117 in humans (respective position in bacteria: G128 [25]) was associated with severe cases of citrullinemia [25]. The glycine residue at this position interacts directly with ATP in the nucleotide binding domain and the serine substitution blocks ATP binding [7]. This glycine to serine substitution represents an ideal point mutation to evaluate the impact of an inactive enzyme.

We report here studies that confirm the enzyme activity of ASS in *L. donovani* (LdASS) and its involvement in parasite pathogenesis by examining the impact of expression of an inactive ASS mutant in the parasite during infection of mice.

## Materials and Methods

### Ethics statement

For animals use, procedures used were reviewed and approved by the Animal Care and Use Committee, Center for Biologics Evaluation and Research, Food and Drug Administration. The CBER ACUC follows “The Guide for the Care and Use of Laboratory Animals,” 8th edition by the Institute for Laboratory Animal Research.

### Parasite and bacterial strains

The *L. donovani* cloned line designated by the World Health Organization as MHOM/SD/62/1S-C12D (Ld1S2D) was used in all the experiments [26]–[27], and the over expressing parasites were derived from that strain. Promastigotes and the axenic amastigotes were grown and harvested as described previously [27]. Top10 and BL21 (DE3) pLysS *E. coli* competent cells (Invitrogen) were used to maintain the plasmid constructs and for protein expression, respectively.

### Preparation of cells lines over expressing ASS

COOH terminal wild-type (LdASS^WT^-V5His) and site-directed mutant G128S (LdASS^G128S^-V5His) ASS were created using the pCRT7-CT/TOPO plasmid vector (Invitrogen) which adds a V5 epitope and hexahistidine tag to the C terminus of the recombinant protein. Briefly, the ASS coding sequence (accession number JQ015382 and GenBank identity: AFD04704.1) was PCR amplified from *L. donovani* genomic DNA using F1/R1 primer pairs (Supplemental table S1). In order to introduce the mutation G128S (Gly^128^→Serine), we first created two short PCR fragments using F2/R1 and F1/R2 primer pairs. Those two PCR products were mixed with primer pairs F1/R1 in a PCR reaction that generated a full length ASS open reading frame with the G128S mutation. The NH_2_ constructs were generated by PCR amplification of the WT and G128S coding sequences from the pCRT7 plasmids using primers F3/R3 and ligation into pEXP-5-NT/TOPO (Invitrogen).

Constructs for the over expressing parasites were prepared by amplification from the corresponding pEXP-5-NT or pCRT7-CT plasmids using respectively the F4/R4 or F5/R5 primer pairs thus copying the ASS coding sequence with the V5 and hexahistidine tags at the N terminus or C terminus as well as adding *SpeI* restriction sites followed by cloning into pCR/2.1, used to transform competent *E. coli*. The purified plasmids were digested; *SpeI* fragments gel purified and ligated into the pKSNeo, an episomal expression vector used to transfect *Leishmania* parasites [28]. All constructs were verified by automated DNA sequence analysis.

*Ld1S2D* parasites were transfected and cloned as described previously [29]. Briefly, logarithmic phase promastigotes were re-suspended at 1×10^8^ cells/mL in electroporation buffer and 5×10^7^ cells were transfected with 20 µg of plasmid DNA by electroporation with the Genepulser apparatus using settings of 0.45 kV and 500 µF (BioRad, Hercules, CA USA). Recombinant *L. donovani* promastigotes were selected on M199 media containing G418 at 20 µg/mL (SigmaAldrich).

For Western blots, parasites were washed once with ice-cold PBS and re-suspended in RIPA buffer. Proteins were separated using SDS-PAGE gels, transferred to nitrocellulose membrane (Pall corporation, Pansacola, FL), and blocked overnight in 5% non fat dried milk in Tris Buffered Saline +0.05% Tween-20 (TBS-T). Blots were incubated for 1 h with either affinity purified rabbit anti-LdASS antibody (1∶2,500) [4] or rabbit anti-V5 antibody (1∶2,000) or normal rabbit serum NRS (1∶2,000) or mouse monoclonal anti-tubulin antibody (Sigma, St. Louis, MO; catalog number: T9026) (1∶2,000), followed by goat anti-rabbit or anti-mouse IRDye 800 cw (Li-COR, Biosciences) secondary antibody at a 1∶10,000 dilution. Immunoblots were developed using the Odyssey Infrared Imaging System (Li-COR). The anti-V5 antibody was produced by cloning a random DNA fragment into the pCRT7-CT plasmid in-frame with the V5 (AAG GGC AAT TCG AAG CTT GAA GGT AAG CCT ATC CCT AAC CCT CTC CTC GGT CTC GAT TCT ACG CGT ACC GGT/KGNSKLEGKPIPNPLLGLDSTRTG) and hexahistidine (CAT CAT CAC CAT CAC CAT/HHHHHH) sequences. Bacterially expressed recombinant protein was purified by Ni-NTA-agarose according to the manufacturer's protocol (Qiagen, Inc. Valencia, CA). Recombinant protein solutions were injected into New Zealand White rabbits according to the company protocol (Spring Valley Laboratories, Woodbine, MD). The antiserum was used to bind Western blots containing whole *Leishmania* parasite lysate and showed no reactivity with any *Leishmania* proteins (data not shown). However, the antiserum is potently reactive in Western blots and ELISA with all proteins tested that contain the V5 epitope tag.

### ASS pull down and activity assay

Amastigotes from each cell line were suspended in lysis buffer (150 mM NaCl, 1 mM EDTA, 10 mM Tris–HCl, pH 7.5, 1% Nonidet P-40 with protease inhibitor cocktail) (10^8^ parasites in 125 µl lysis buffer per reaction) for 1 h on ice. The cleared lysate obtained by centrifugation for 20 min at 10,000 rpm and corresponding to 1×10^8^ parasites, was quantified using the bicinchoninic acid (BCA, Pierce, Rockford, IL, USA) method [30] and used to normalize the assay. Lysates were then incubated with Sepharose for 10 min to clear non-specific binding and then incubated with 50% slurry (25 µl) of Ni^2+^-NTAagarose beads (Amersham Pharmacia) and incubated overnight at 4°C. The Ni^2+^-NTA beads were then washed three times with lysis buffer containing 300 mM NaCl. Bound proteins were analyzed by SDS-PAGE or used for the ASS activity assays, which were a modified version of the assay described by Guerreiro et al [31]. Briefly, proteins bound to the bead pellet (12.5 µl per reaction) were resuspended in reaction buffer (20 mM Tris-HCl, pH 7.8, 4 mM ATP, 4 mM citrulline, 4 mM aspartate, 6 mM MgCl_2_, 20 mM KCl, and 0.2 units of pyrophosphatase) in a final volume of 20 µl. Reactions without citrulline and aspartate substrates were also prepared. Reactions were incubated in triplicates at 37°C in 96-well microtiter plates, and then stopped after 30 min by the addition of 300 µl volume of malachite green reagent [32]. Accumulation of phosphate was determined spectrophotometrically at 650 nm, and its concentration was interpolated from a standard curve of inorganic phosphate (Pi). Due to spontaneous release of Pi in the absence of substrates, the concentration of Pi released from these affinity purified proteins or mouse liver extract (MLE: positive control of the reaction) were determined by subtracting the mean of the values found in the absence of substrates from the mean of the values found in presence of substrates. The specific ASS activity was determined using this formula: nmoles of Pi released/quantity of protein/hour.

In order to assess the formation of arginosuccinic acid (ASA) in the conditions of our assay, similar reactions were run in parallel in the absence of pyrophosphatase. After incubation for 1 h at 37°C, the supernatant separated from the bead pellet was subjected to reverse phase high performance liquid chromatography (HPLC). ASA was analyzed by HPLC after pre-column derivatization with *O*-Phthaldialdehyde (OPA) to convert the reaction products to fluorescent derivatives, as described by Portoles and Rubio [33], with some modifications. The HPLC device was an Agilent 1260 Infinity system with an autosampler and a FLD fluorescence detector. The reverse-phase column was Agilent ZORBAX Eclipse XDB-C18 analytical column (4.6×150 mm, 5-Micron). Both the autosampler and the column compartment were set at 22°C during the analysis. Automated sample derivatization in the autosampler was performed using the following injection program: equal volume (10 µl) of the sample and OPA were drawn and mixed in the needle seat for 10 times at high speed, 20 µl of reaction mixture injected 2.0 minutes after mixing. To mimic the convex gradient described in Portoles and Rubio protocol, a sequential change in linear gradient was achieved by increasing solvent B (65% methanol) from 0 to 20% within 23 minutes with the following time segments: 0.5 min at 0%, 1.2 min at 2.5%, 2.5 min at 5%, 4.5 min at 8%, 8.1 min at 11.5%, 15.8 min at 16.5%, and 23 min at 20%. Solvent B was then increased to 100% in 0.5 min and held for 6.5 min for column cleaning. Solvent A (2 V methanol, 2 V tetrahydrofuran and 96 V of 50 mM NaOAc, 50 mM Na2HPO4; pH 7.5) was brought to 100% in 0.5 min and held for 6.5 min to re-equilibrate the column before the next injection.

### Immunofluorescence analysis

For IFA, we used the same protocol described by Furuya et al. [34] except for the slide mounting step. Briefly, *L. donovani* amastigotes were washed in PBS and allowed to attach to Poly-L-lysine glass slides for 20 min. Parasites were fixed in 4% PFA for 5 min, permeabilized with 0.1% Triton X-100 in PBS for 5 min and blocked for 1 h with 10% nonfat dry milk in PBS. The slides were then incubated for 1 h with the affinity purified rabbit anti-LdASS (1∶200) diluted in blocking buffer. After 6 washes in PBS, slides were incubated for 1 h with Alexa Fluor 488 goat anti-Rabbit IgG (1∶200, Invitrogen) as a secondary antibody in blocking solution. For co-localization experiments, biotinylated anti-LdASS (1∶100 dilution), was used with rabbit anti-HGPRT (1∶200) [35], or rabbit anti-LdPEX14 (1∶1,000) [36]. The biotinylation of anti-LdASS antibody was done with purified IgG using the E-Z-Link Iodoacetyl-LC-Biotin kit (Thermo Scientific) according to the manufacture's protocol. Strepatividin-Alexa 488 (1∶1,000) and Alexa fluor 594 goat anti-Rabbit IgG (1∶200, Invitrogen, Molecular probes) were used as secondary antibodies. Affinity purified anti-LdASS was used with guinea pig anti-LdIMPDH (1∶200) [37] for co-localization of ASS with IMPDH. Slides were incubated for 1 h with Alexa Fluor 488 goat anti-Rabbit IgG (1∶200, Invitrogen) and Alexa fluor 594 goat anti-guinea pig IgG (1∶200, Invitrogen) as secondary antibodies in blocking solution. In all cases, a parasite preparation was stained with secondary antibody alone. The captured images showed no cell-associated fluorescence (data not shown). Slides were subsequently washed 6 times with PBS and mounted in Vectashield containing 4′6-diamidino-2-phenylindole (DAPI, VectorLab Inc.) to stain both nucleus and kinetoplast. Cells were examined for fluorescence under the microscope (Nikon Eclipse TE2000-U), and 0.3 micron thick optical sections were captured and identical slices from all the channels were processed with Open lab 5.2 software (Perkin Elmer, Waltham, MA) to generate deconvoluted images. The focal plane chosen in all the images was in the middle of the cells. The images were further processed using Adobe Photoshop 5.5 (Adobe Systems Inc., Mountain View, CA) [26].

### Expression and purification of recombinant ASS proteins and LdPEX5-ASS binding assays

*Escherichia coli* BL21 (DE3) plysS cells (Invitrogen) transformed with pEXP5-V5HisLdASS^WT^, pEXP5-V5HisLdASS^G128S^, pCRT7-LdASS^WT^V5His or pCRT7-LdASS^G128S^V5His were grown in LB broth with 100 µg/mL ampicillin and 34 µg/mL Chloramphenicol to an absorbance of 0.8 at 600 nm and then induced with 0.1 mM isopropyl thiogalactoside for 4 h at 37°C with vigorous shaking. Bacterial cultures were harvested and then the recombinant protein was purified from the cell pellet under non-denaturing conditions using Ni-NTA agarose (QIAGEN) following the manufacturer's instructions. Column eluates containing each of the LdASS forms were dialyzed against PBS and stored at −80°C. The PTS1 receptor protein LdPEX5 and the glycosomal PTS1 protein inosine monophosphate dehydrogenase (LdIMPDH) were expressed and purified to homogeneity as previously described [37]–[38].

ELISA-based LdPEX5–LdASS interaction assays were done as previously described [39]. Briefly, plates were coated with 100 µL of 10 µg/mL protein in PBS at 4°C for 16 h. Unbound proteins was discarded and plates were blocked with 200 µL of 2% bovine serum albumin in PBS and then incubated with decreasing concentration (0.01–400 nM) of purified LdPEX5 for plates coated with LdIMPDH and the LdASS variants and the bound LdPEX5 was detected using anti-LdPEX5 polyclonal antisera [38]. Alternatively, plates were coated with LdPEX5 (100 µL of 10 µg/mL protein in PBS) and then incubated with increasing concentrations of with LdIMPDH and the LdASS variants (0.01–200 nM) and the bound PTS1 proteins were visualized using by indirect ELISA using either the anti-LdIMPDH or anti-V5 primary polyclonal antibodies.

Immunoprecipitaion with anti-LdPEX5 antibody or preimmune sera was done as previously described using Ld1S2D axenic amastigotes [40]. For Western blot, the membrane was incubated with anti-LdASS antibody (1∶2,000) for 1 h followed by an 1 h incubation with Mouse anti-rabbit IgG (light-chain specific) (1∶2,000) (Cell signaling) and then 1 h with anti-mouse IRDye 800 cw (1∶10,000) (Li-COR, Biosciences). The reverse Pull-Down was done using nickel agarose beads (since the affinity purified anti-LdASS does not perform well in immunoprecipitation) and V5His-LdASS^WT^ parasites and the anti-LdPEX5 antibody (1∶1,000) or anti-LdASS (1∶2,500) were used for the Western blot. Immunoblots were developed using the Odyssey Infrared Imaging System (Li-COR).

### Subcellular fractionation, Western blotting and protease treatment

Glycosome isolation and protease K (PK) treatment were modified versions of the protocol described by Pilar et al, 2008 [23]. Briefly, 3×10^10^ amastigotes from the V5His-LdASS^WT^ cell line were harvested, washed once in cold PBS and once in hypotonic buffer and finally lysed in 8 mL hypotonic buffer for 5 min on ice. A cell homogenate was obtained by expulsion of the cells through a 27 gauge needle 10 times. The lysate was made isotonic by adding 2 mL isotonic buffer and then cleared at 5000× g for 10 min at 4°C and the supernatant collected as the post nuclear fraction. This fraction was centrifuged at 45,000× g for 45 min to obtain the crude organelle fraction containing glycosomes in the pellet and the cytosol in the supernatant. The pellet was used either for protease K (PK) treatment or for fractionation over a sucrose gradient. For PK treatment, the pellet was resuspended in PBS and treated in the presence or absence of 1% triton with 150 µg/ml PK for 1 hour on ice. The reaction was stopped by precipitation with 20% TCA and then analyzed by Western blot. For the sucrose gradient, the crude organelle pellet was resuspended in 25 mM Hepes-NaOH pH 7.4, and centrifuged for 2 min at 5,000 rpm. The resulting supernatant was layered on a 70-20% sucrose gradient prepared by layering decreasing concentrations of sucrose fractions in 25 mM Hepes-NaOH, pH 7.4. Differential centrifugation was performed for 6 h at 221,900× g using a SW41 rotor at 4°C. The gradient was fractionated from the top in 0.5 mL fractions that were TCA precipitated and used for western blotting. The fractions were separated by SDS-polyacrylamide gel (12%) and transferred to PVDF membrane. The membranes were probed either with rabbit anti-V5antibody (1∶2,000), rabbit anti-HGPRT (1∶4,000), as a marker for glycosomes or rabbit anti-*T. brucei* -TatD (1∶1,000) as a marker for cytosolic proteins [41], washed then probed with Goat anti-Rabbit IRDye 800 cw (1∶10,000) (Li-COR, Biosciences). Immunoblots were developed using the Odyssey Infrared Imaging System (Li-COR). Estimates of the quantity of specific proteins labeled by antibodies on Western blots were obtained from the Odyssey infrared image using the associated software. The quantity of each protein measured in this fashion was divided by the portion of each sample that was loaded on the gel to estimate the total portion in each fraction.

### Animals and infection

Six- to eight-week-old female BALB/c mice from the National Cancer Institute were used in the experiments. Procedures used were reviewed and approved by the Animal Care and Use Committee, Center for Biologics Evaluation and Research, Food and Drug Administration. BALB/c mice were inoculated via tail vein with 3×10^6^ metacyclic stage parasites transfected with the vector control, V5His-LdASS^WT^, or V5His-LdASS^G128S^ expression plasmids. Infective-stage metacyclic promastigotes of *L. donovani* were isolated from stationary cultures by density gradient centrifugation as described previously [2]. Five weeks post-infection, all the mice were sacrificed and parasite burdens in the liver and spleen were measured by the serial dilution method as previously described [2]. Western blot analysis of recovered parasites was done as previously described in section 2.2.

For the statistical analysis, differences in the number of parasites between groups of mice were analyzed using one-way analysis of variance, followed by Tukey multiple comparison test. The two-sided test was used for multiple comparisons with the level of significance at 0.05.

## Results

### Identification of a homolog of argininosuccinate synthase in *L. donovani*

Using the microarray technique, we previously identified several genomic clones that were differentially expressed between the promastigote and axenic amastigote forms of *LdCen^−/−^* [4]. For this study, we selected the open reading frame (ORF) that encodes a putative argininosuccinate synthase (ASS) for a complete characterization. The ASS's ORF is 1257 bp in length and is present at a single locus on the *L. donovani* genome (chromosome 23). It encodes a predicted 419 amino acid protein with a predicted molecular weight of 46 kDa and an isoelectric point of 5.8 (http://www.bioinformatics.org/sms2/protein_iep.html). Cloning and sequencing of the LdASS ORF from the *Ld1S2D* strain (GenBank: AFD04704.1) revealed that the predicted LdASS protein is identical to *L. infantum* LinJ.23.0300 in the annotated genome database [http://tritrypdb.org/tritrypdb/] and to CBZ34226.1 in the recently annotated and published *L. donovani* BPK282A1genome. The deduced amino acid sequence of the LdASS ORF (Supplemental Figure S1) is 59.7% similar to human ASS (hASS) and contains (i) a nucleotide binding domain, (ii) the synthetase domain (iii) an oligomerization tail region and (iv) a putative glycosomal targeting signal (aa 416–418) at its C-terminal end. The glycine at position 117 in the human sequence, when substituted with serine, results in a severe form of citrullinemia [25]. A corresponding glycine is conserved at position 128 in the *L. donovani* amino acid sequence.

**Figure 1:**
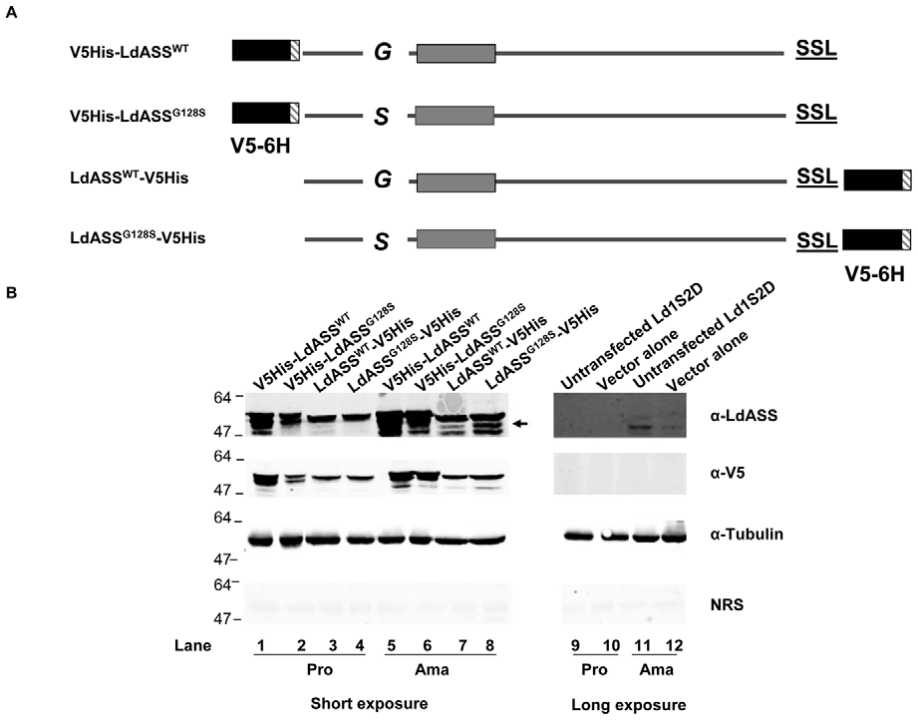
Generation of cell lines over expressing ASS. (A) Diagram of recombinant LdASS constructs used in this study. The black boxes represent the V5 epitope and grey striped boxes represent hexahistidine tags. The solid gray box indicates the enzyme active site and G or S indicates the residue at position 128. SSL indicates the 3 residues at the C terminus that have been described as a PTS1 glycosomal targeting signal. V5His-LdASS^WT^ and LdASS^WT^-V5His: ASS native sequence with epitope tags at the amino terminus and carboxy terminal respectively. V5His-LdASS^G128S^ and LdASS^G128S^-V5His: ASS native sequence with a serine substitution at glycine 128 and epitope tags at the amino terminus and carboxy terminal respectively. (B) Western blot analysis: Equal amounts of parasite lysates (10^6^ cell equivalents/lane) of untransfected *Ld1S2D* or cells stably transfected with empty vector or constructs shown above in each lane, taken at 2 different stages (Pro-promastigotes and Ama- amastigotes) were separated by SDS page and transferred to nitrocellulose membranes. The Western blots were probed with anti-LdASS, anti-V5, anti-Tubulin antibodies or pre immune sera (left panel: short exposure and right panel: long exposure). The arrow indicates the endogenous LdASS.

Functional characterization of genes in *Leishmania* often involves creation of targeted gene deleted parasites and analysis of the effect of gene loss on parasite survival and infectivity. Such a strategy was not successful in our study since LdASS is present on chromosome 23 that has at least 3 copies in the *L. donovani* 1S2D genome (Personal communication, Peter Myler). Future studies will focus on the deletion of all the copies of the *L. donovani* ASS gene to address this question. Alternatively, to characterize LdASS and its function, we employed a dominant negative approach. Four cell lines were created expressing the wild type or mutant form of LdASS (G128S) with V5epitopes and hexasitidine tags at either the N-terminal (V5His-LdASS^WT^, V5His-LdASS^G128S^) or C-terminal (LdASS*^WT^*-V5His, LdASS*^G128S^*-V5His) ends (Fig. 1A). All four exhibited similar growth kinetics *in vitro* either in promastigote or axenic amastigote forms (data not shown). Immunoblot analysis of total cell lysates from these transfectants cultured as the promastigote or axenic amastigote forms indicated that the exogenous protein reacted with both the anti-LdASS and anti-V5 antibodies, was present in excess of the native protein in both life cycle stages, the recombinant form of LdASS was detected predominantly as a doublet that migrated at 47 kDa and 49 kDa, and the expression resulted in extra bands (Fig. 1B; V5His-LdASS^WT^ (lanes 1, 5); V5His-LdASS^G128S^ (lanes 2, 6); LdASS^WT^-V5His (lanes 3, 7); LdASS^G128S^-V5His (lanes 4, 8)). Longer exposure time was required to reveal native ASS protein (Fig. 1B; lanes 9–12). The endogenous LdASS both in non-transfected and vector alone transfected parasites migrated at 48 kDa, reacted with the anti-LdASS antibody but not with the anti-V5 and was exclusively expressed in the amastigote form (Fig. 1.B; lanes 11 and 12). The anti-tubulin blot showed that the same quantity of lysates was loaded in each lane on the gel (Fig. 1B). Parasite lysates probed with normal rabbit serum (NRS) did not show any reactivity supporting the specificity of the reactive anti-sera.

### The *Leishmania* homolog has argininosuccinate synthase activity and the G128S mutation abrogates the activity

ASS is a key enzyme in the Urea cycle that catalyzes the ATP-dependant conversion of citrulline and L-aspartate to argininosuccinate (ASA) [8]. In order to verify whether LdASS was functional, we used an *in vitro* assay coupling citrulline with aspartate as substrates in the presence of ATP and measured the release of Pi and assessed in parallel ASA formation by reverse phase HPLC as described by Portoles [33].

**Figure 2:**
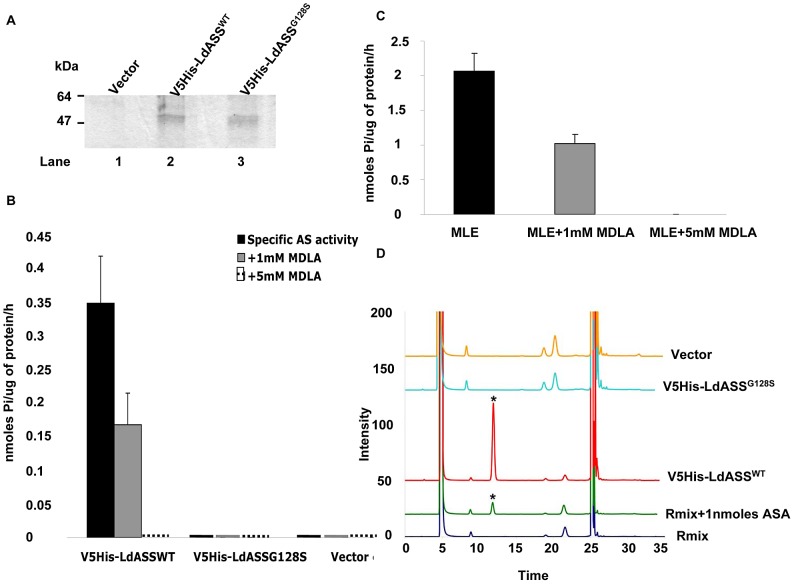
ASS activity assay. (A) Sample of recombinant ASS proteins affinity purified from 10^8^ cells of vector control (lane 1), V5His-LdASS^WT^ (lane 2) and V5His-LdASS^G128S^ (lane 3) over expressing parasites using Ni-NTA resin beads, analyzed on an SDS gel and visualized with Gel code blue staining. (B) ASS activity measured by release of Pi in the presence of aspartate and citrulline substrates, obtained with *Leishmania* recombinant ASS proteins purified from the over expressing cell lines, in the absence (black bars), or presence of 1 mM (grey bars) or 5 mM of inhibitor (interrupted line). (C) Activity determined for Mouse Liver Extract (MLE) as a positive control for the assay. (D) HPLC analysis: Fluorescence intensity of OPA labeled components of ASS enzyme reaction. Overlay of elution profiles showing resolution of ASA (blue line: reaction mix, green line: 1 nmoles of ASA mixed with the reaction mix, red line: pulled down protein from V5His-LdASS^WT^ cell line and incubated in presence of the reaction mix, turquoise line: pulled down protein from V5His-LdASS^G128S^ cell line incubated in presence of the reaction mix, orange line: pull down from vector control incubated in presence of the reaction mix. The star indicates the ASA peak. The graphs shown are representative of 3 replicates of the assay.

Preliminary studies showed that the endogenous enzymatic activity of ASS in *L. donovani* is very low; hence we were not able to detect it (data not shown). In order to demonstrate the activity of LdASS in the parasite, we used the two cell lines over expressing LdASS with an N-terminal tag epitope for the *in vitro* assay. *Leishmania* parasites (1×10^8^) expressing V5His-LdASS^WT^ or V5His-LdASS^G128S^ (point mutant) were used as the starting material to affinity purify these proteins from the respective transfectants by metal affinity chromatography using Ni^2+^-NTA agarose. A protein stained SDS gel showed that a band of the same intensity corresponding to the exogenous ASS was pulled down from both transfectant cell lines (Fig. 2A, lanes 2 and 3). As expected, no visible protein band was purified from the cells transfected with the empty vector control (Fig. 2A, lane 1).

Enzymatic assays of the affinity purified proteins using an optimized ASS activity assay that monitored the release of inorganic phosphate (Pi) from ATP showed that V5His-LdASS^WT^ had a specific activity of 0.35 nmoles Pi/ug of protein/h (Fig. 2B). Consistent with the accumulation of citrulline in individuals with the human ASSG117S mutation; the V5His-LdASS^G128S^ protein did not have any activity. The vector control showed there was no background activity in this assay. The performance of the Pi release assay was validated using mouse liver extract (MLE) (Fig. 2C), which showed abundant activity of the mammalian enzyme in the crude extract.

In order to further confirm that the observed activity was ASS specific, we tested the capacity of methyl-D-L-Aspartic acid (MDLA), a specific ASS inhibitor [42] that acts as a competitor for aspartic acid, at a concentration of 1 mM and 5 mM to inhibit ASS activity. At 1 mM MDLA, LdASS activity was reduced by ∼60%, while at 5 mM MDLA, the catalytic activity was completely abolished (Fig. 2B and C). Although effective in abolishing enzyme activity *in vitro*, when this MDLA was added to parasite culture medium at a final concentration of 5 mM it had no effect in decreasing parasite growth (data not shown).

In order to demonstrate that the LdASS catalyzed reaction is producing the correct product, we checked for the presence of ASA by reverse phase HPLC. The addition of 1 nmoles of commercial ASA to the reaction mix yielded a peak at 11.9 min, not seen with the reaction mix alone (Fig. 2D, indicated by asterisk). A peak with the same retention was also seen in the products of a reaction catalyzed by the V5His-LdASS^WT^ pulled-down protein in the absence of pyrophosphatase. Migration of the peak corresponding to ASA in V5His-LdAssWT pull down enzymatic activity analysis confirms the identity of the product being made by LdASS. Furthermore, no peak was obtained when the vector control or point mutant protein pull-downs were used as the source of enzyme (Fig. 2D).

Taken together, these results suggest that LdASS has argininosuccinate synthase activity and that a single amino acid substitution in the ATP binding site in the recombinant LdASS results in loss of enzyme activity.

### ASS substrates can not substitute for arginine in *Leishmania* culture

Given that the mammalian homolog of ASS is involved in the *de novo* arginine biosynthesis [15], we examined the growth of *Ld1S2D*, as well as the LdASS over expressing parasites, in chemically defined media (serum free M199 media, i) lacking 5 amino-acids, in pathways that feed into arginine biosynthesis: Arg, Ala, Asp, Glu and Gln), ii) supplemented with arginine in the absence of citrulline and aspartate, or iii) supplemented with citrulline and L-aspartate in the absence of arginine. Similar growth kinetics were obtained for *Ld1S2D* as well as for the cells over expressing wild type or mutant ASS. The parasites cultured in the presence of arginine grew well, however, the parasites grown in the presence of aspartate and citrulline without arginine failed to grow, suggesting that these 2 substrates alone did not substitute for arginine (supplemental Figure S2). This result is consistent with the absence of an argininosuccinate lyase from the *Leishmania* genome, the enzyme responsible for breaking down argininosuccinate into arginine and fumarate.

### Toward identifying the subcellular localization of LdASS

**Figure 3:**
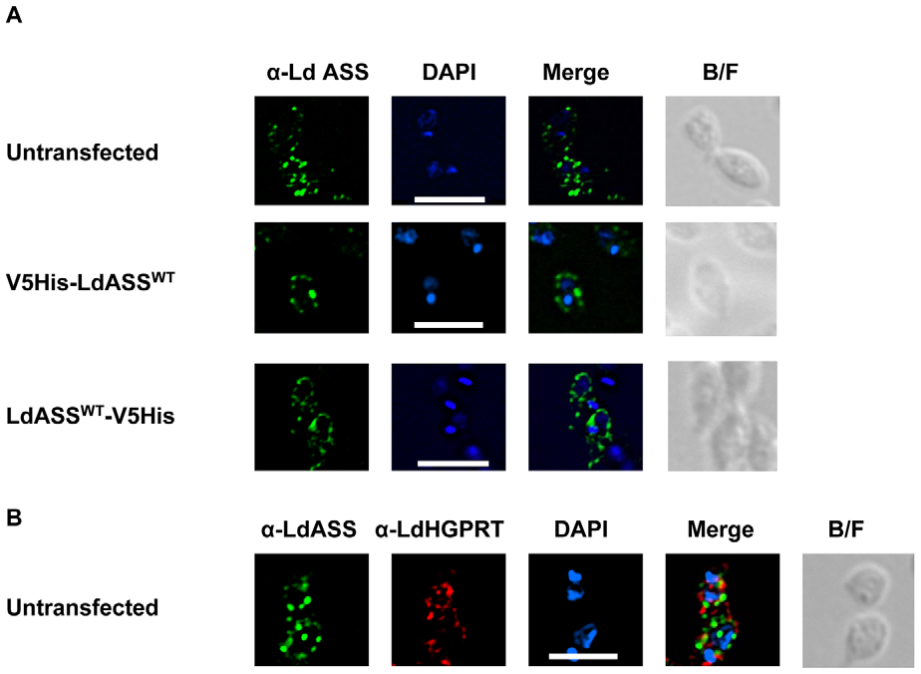
Immunofluorescence analysis of LdASS. *L. donovani* amastigote cells were stained with affinity purified antibody against LdASS as primary, and Alexa488-conjugated anti-rabbit IgG (green) as secondary antibodies. Panel A: upper row: untransfected parasites, middle row: V5His-LdASS^WT^ transfected parasites, lower row: LdASS^WT^-V5His transfected parasites. Panel B: untransfected parasites labeled with biotinylated anti-LdASS (green) and anti-HGPRT antibodies (red). The nuclei and kinetoplast are stained with DAPI (blue). The merge of the images is shown in the column labeled “Merge” in the figure. The right panel shows the bright field images. The white bar represents the scale: 10 µm.

Having shown that LdASS has enzymatic activity, to gain a better understanding of its physiological role we investigated the subcellular localization of this enzyme as an indication of the pathways in which ASS might be involved. Analysis of the deduced amino acid sequence revealed that LdASS has a C-terminal Ser-Ser-Leu amino acid sequence (PTS1 signal), a targeting signal that directs proteins for glycosomal import [22] (Fig. S1). To determine whether LdASS has glycosomal localization, the antibodies raised against *L. donovani* ASS were used for immunofluorescence studies. IFA analysis showed both untransfected *Ld1S2D* or *Ld1S2D* parasites over expressing LdASS (V5His-LdASS^WT^ and LdASS^WT^-V5His cell lines) had a punctate pattern (Fig. 3A) suggesting that LdASS may be localized in an intracellular organelle. No cell-associated fluorescence was seen with the cells stained with the secondary antibody only (data not shown).

To identify the organelle to which LdASS trafficked, colocalization experiments staining parasites with antibodies specific for the *L. donovani* HGPRT, a known glycosomal marker [35], and anti-LdASS, showed that LdASS and LdHGPRT were not present in the same subcellular location (Fig. 3B) and suggested that LdASS may be targeted to a new subset of glycosomes or non-glycosomal vesicles. It is important to note that the parasites stained in the same conditions with either of the secondary antibodies alone did not show any cell-associated fluorescence (data not shown). A similar IFA pattern was also observed with the point mutant V5His-LdASS^G128S^ (data not shown).

### LdASS interactions with the glycosomal trafficking machinery

To gain further insights into whether the PTS1 signal of LdASS plays a role in targeting to putative glycosome-like vesicles, we examined the interaction of LdASS with the glycosomal import machinery. In *Leishmania*, newly synthesized proteins containing PTS1 signals are bound by the receptor peroxin 5 (LdPEX5) in the cytosol. The import of PTS1 proteins into the glycosome requires binding of the PTS1-laden LdPEX5 receptor to the membrane-associated protein LdPEX14 to facilitate translocation of PTS1 proteins into the lumen of these organelles [23].

**Figure 4:**
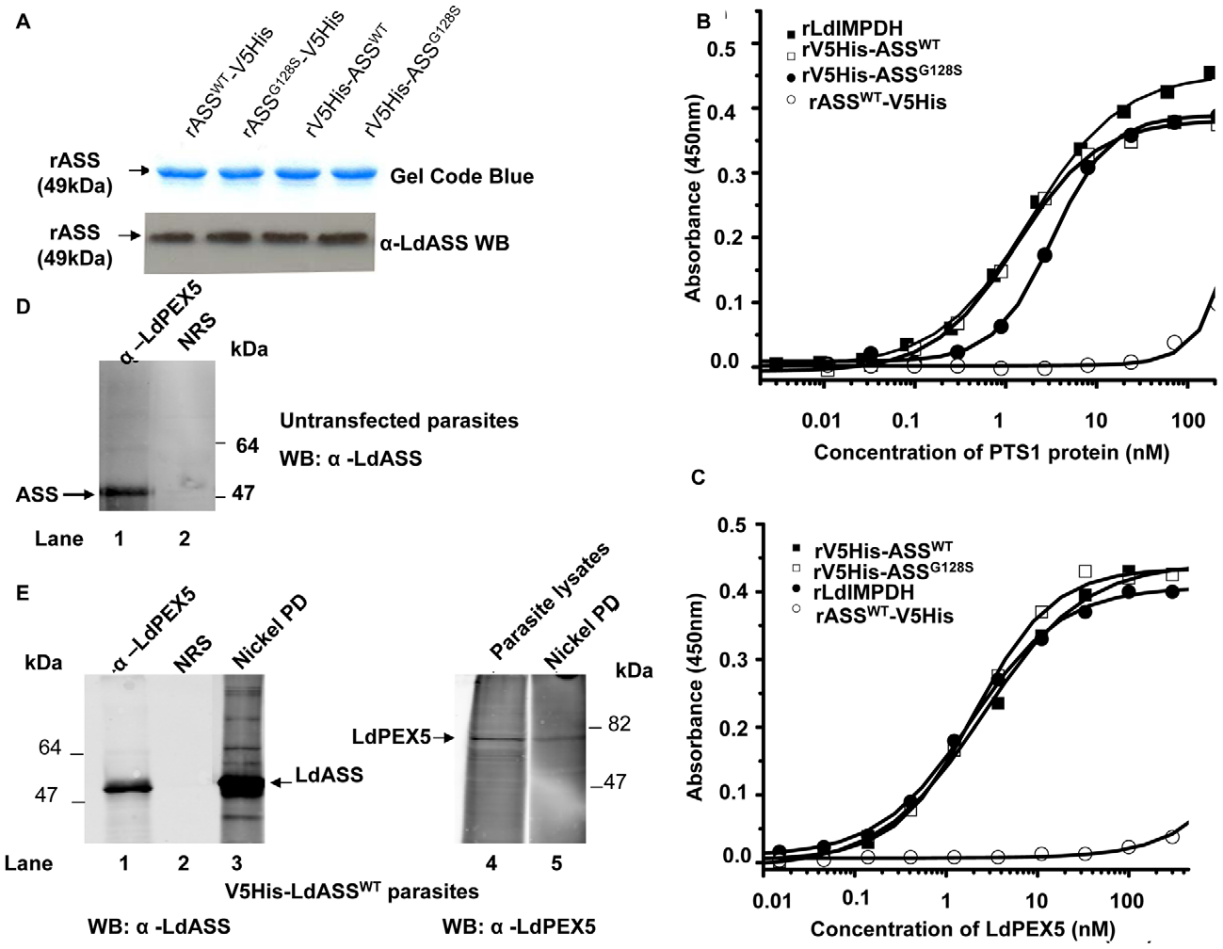
LdAS-LdPEX5 interactions. (A) SDS gel of recombinant ASS proteins expressed in bacteria and purified over Ni-NTA resin beads, stained with Gel Code Blue (Upper panel). The lower panel corresponds to the anti-LdASS Western blot of the recombinant proteins. (B) Microtiter plates were coated with decreasing concentrations of recombinant LdASS forms or LdIMPDH and then incubated with LdPEX5. Bound LdPEX5 was quantified by an indirect ELISA using anti-LdPEX5 antiserum. Each assay was performed in triplicate and the average absorbance values were plotted as a function of the log of the PTS1 protein concentration using the ORIGIN 7.0 software. (C) Microtiter plates were coated with decreasing concentrations LdPEX5 and then incubated with PTS1 proteins. Bound PTS1 proteins were quantified by an indirect ELISA using anti-V5 or LdIMPDH antisera. (D) LdPEX5 pull-down assay: protein lysates from *Ld1S2D* were used in a co-immunoprecipitation reaction with Anti-LdPEX5 antibody or preimmune sera (NRS) and the immuno-blot was bound with anti-LdASS antibody. (E) Nickel agarose pull-down (PD): Left panel: protein lysates from V5His-LdASS^WT^ were used in a co-immunoprecipitation reaction with Anti-LdPEX5 antibody (lane 1) or preimmune sera (NRS) (lane 2) or for nickel agarose PD (lane 3) and the immuno-blot was bound with anti-LdASS antibody. Right panel: immuno-blot using cleared lysates (lane 4) or nickel agarose PD proteins (lane 5) was bound with anti-LdPEX5 antibody.

To analyze the interactions in sorting and trafficking, we first analyzed the LdPEX5-LdASS interaction and second we examined the potential colocalization of LdASS with LdPEX14. To this end, we used a bacterial expression system to produce the recombinant LdASS proteins in 4 different forms: the same coding sequences expressed in *Leishmania* described above (Fig. 1A) were ligated into bacterial expression constructs. Recombinant LdASS^WT^ and LdASS^G128S^ proteins with N- or C-terminal tags were purified using Ni^2+^-NTA agarose affinity matrix. SDS-PAGE analysis showed the correct sizes (49 kDa) of all of the purified recombinant epitope tagged LdASS proteins (Fig. 4A, upper panel) that reacted with anti-LdASS antibody (Fig. 4A, lower panel). The purified proteins were then used to assess the capacity of the glycosomal PTS1 targeting receptor protein LdPEX5 to recognize the PTS1 tripeptide signal Ser-Ser-Leu on the LdASS proteins using a modified enzyme linked immunoadsorbant assay (ELISA) [39]. In the first experiment, microtiter plates were coated with the three variants of the epitope tagged LdASS and the LdIMPDH, a glycosomal enzyme that contain the C-terminal tripeptide PTS1 targeting signal Ala-Lys-Met, as positive control [37]. The data showed that LdPEX5 bound LdIMPDH and the N-terminal tagged LdASS proteins (both wild type and mutant) with comparable affinities that had a apparent dissociation constant (K_d_) of ∼5 nM (Fig. 4B) which agrees closely with binding constants reported for LdPEX5 [39]. In contrast, no significant LdPEX5 binding was detected with the proteins (both wild type and mutant) in which the C-terminal PTS1 signal sequence was modified by the addition of epitope tags (Fig. 4B, data for rLdASS^G128S^-V5His, not shown). Comparable nanomolar binding affinities were obtained for the LdPEX5-V5His-LdASS and LdPEX5-LdIMPDH interactions using a constant amount of LdPEX5 immobilized on the microtiter plates and varying the level of V5His-LdASS or LdIMPDH in the binding mixture (Fig. 4C). Again no LdASS-V5His binding to LdPEX5 was detected. These experiments show that the tripeptide Ser-Ser-Leu is a bona fide PTS1 signal sequence recognized with high affinity by LdPEX5. Moreover, these experiments confirm that the Ser-Ser-Leu sequence must be located at the C-terminus of the protein to be bound the PTS1 receptor.

In order to confirm that this interaction also takes place *in vivo*, we performed a LdASS/LdPEX5 co-immunoprecipation assay. Axenic amastigote lysates of Ld1S2D were incubated with anti-LdPEX5 antibodies and the bound proteins separated on an SDS gel, transferred to nitrocellulose and the immunoblot developed with anti-LdASS. Figure 4D shows that the LdPEX5 antibody immunoprecipitate contains an anti-LdASS reactive band of 49 kDa corresponding to ASS (Fig. 4D, lane 1). Pull down assays with preimmune sera did not show any immunoreactivity (Fig. 4D, lane 2). It is appropriate to perform a reciprocal IP using anti-LdASS antibody, to unequivocally show the LdPEX5-LdASS interaction. However, the anti-LdASS antibody does not perform well in immunoprecipitation (data not shown). For that reason, we performed a nickel agarose pull-down (PD) from V5His-LdASS^WT^ over expressing parasites. Immunoprecipitation using anti-LdPEX5 or nickel agarose PD from V5His-LdASS^WT^ over expressing parasites (Fig. 4E, lanes 1 and 3) pulled down substantial amounts of LdASS from these cells. Moreover, in the reciprocal experiment, a band corresponding to LdPEX5 was detected on the Western blot containing the nickel agarose PD material bound with the anti-LdPEX5 antibody (Fig. 4E, lane 5) comparable to PEX5 in parasite lysate (Fig. 4E, lane 4).

Demonstrating that ASS binds to the carrier protein LdPEX5 led us to question whether ASS would be carried to the membrane of the glycosome to interact with LdPEX14.

**Figure 5:**
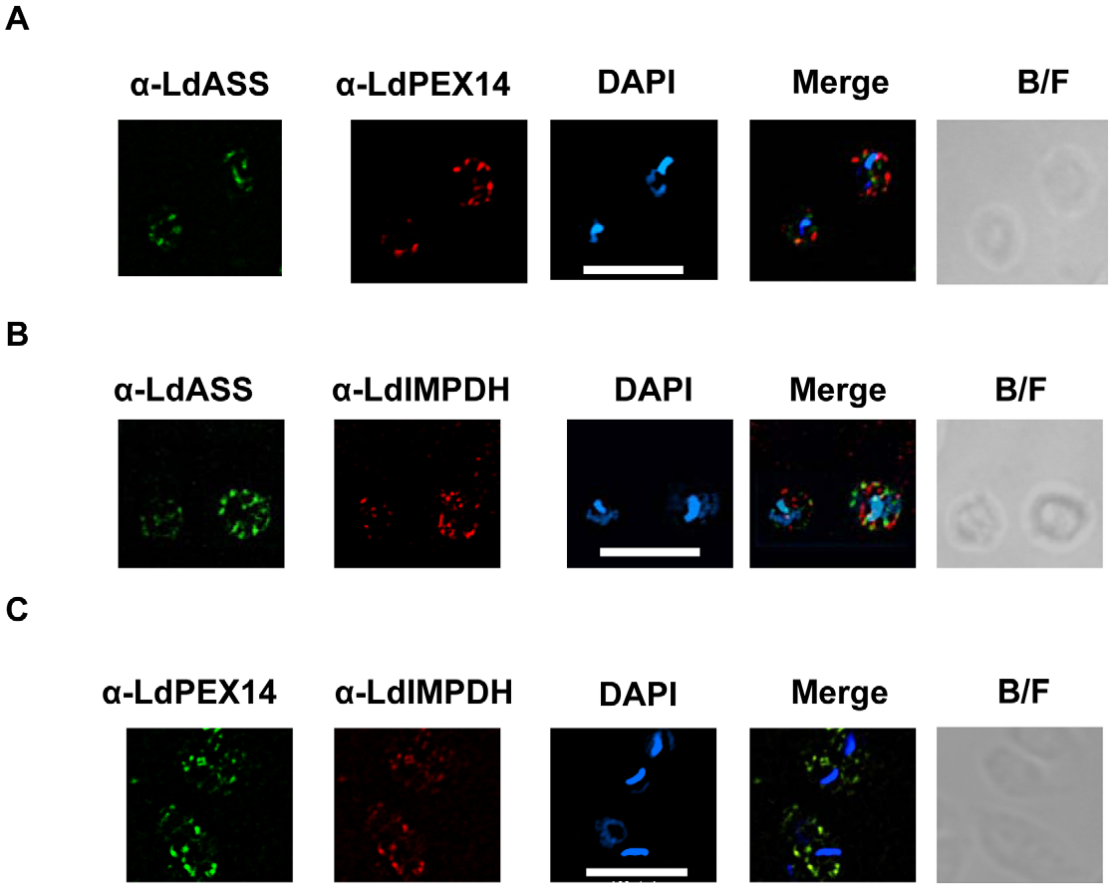
Comparison of LdASS localization with LdPEX14 and LdIMPDH. (A) *L. donovani* amastigotes were stained with antibodies against LdPEX14 and biotinylated anti-LdASS antibodies as primary, then with Alexa568 (red)-conjugated anti-rabbit IgG and Streptavidivin FITC as secondary. The nucleus and kinetoplast were stained with DAPI. (B) *L. donovani* amastigotes were stained with antibodies against affinity purified LdASS and guinea pig anti-LdIMPDH antibodies as primary, then with Alexa488 (red)-conjugated anti-rabbit IgG and Alexa564 (red)-conjugated anti-Guinea-pig IgG as secondary. The nucleus and kinetoplast were stained with DAPI. (C) *L. donovani* amastigotes were stained with rabbit anti-LdPEX14 and Guinea pig anti- LdIMPDH as primary, and Alexa488 (green)-conjugated anti-rabbit IgG and Alexa568 (red)-conjugated anti-guinea pig IgG as secondary antibodies. The nucleus and kinetoplast were stained with DAPI. The merge of the images is shown in the column labeled “Merge”. The right panel shows the bright field images. The white bar represents the scale that corresponds to 10 µm.

IFA performed using anti-LdASS and anti-LdPEX14 antibodies with non-transfected *L. donovani* showed that LdASS localized to small cellular structures that were distributed throughout the cell body. These structures however, did not co-localize with glycosomal membrane associated protein LdPEX14 [36] (Fig. 5A). As another example of a PTS1 containing glycosomal protein, IMPDH localization was compared to LdASS. Affinity purified anti-LdASS did not co-localize with guinea pig anti-LdIMPDH (Fig. 5B). However, similar co-localization experiments performed using anti-LdIMPDH and anti-LdPEX14 antibodies confirmed that both of these proteins were present in the glycosome (Fig. 5C). These results suggest that although LdASS interacts with some of the glycosomal import machinery, it is localized in a subcellular structure that is different from the glycosomes.

### Subcellular vesicle isolation

**Figure 6:**
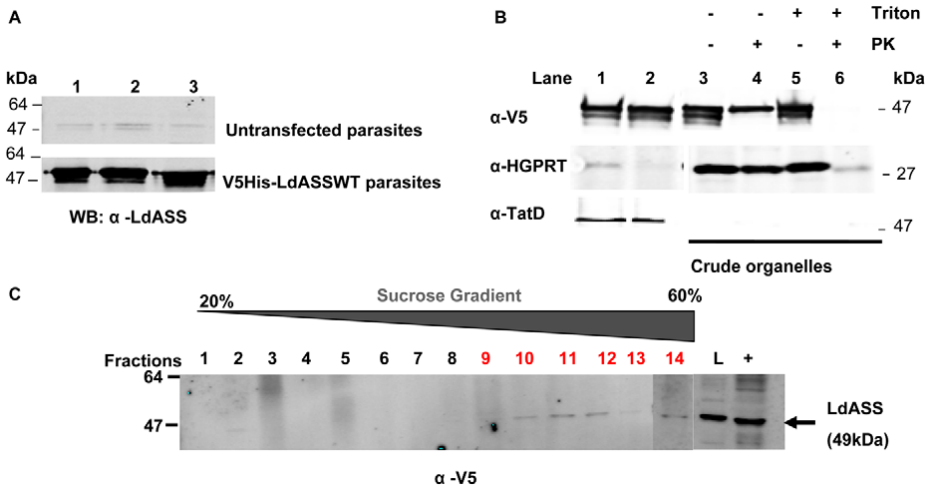
Subcellular organelle fractionation. (A) Cleared lysates (lane 1) from untransfected Ld1S2D parasites (upper panel) or V5-HisLdASS^WT^ over expressing amastigotes (lower panel) were fractionated into cytosol (lane 2) and crude organelles (lane 3) and used for immuno-blot with anti-LdASS antibody. (B) V5-HisLdASS^WT^ over expressing amastigotes were lysed then cleared (lane 1) and fractionated into cytosol (lane 2) and crude organelles (lanes 3–6). Crude organelles were treated with protease K in absence (lane 4) or presence of triton (lane 6). Proteins were separated by SDS page, transferred to nitrocellulose membranes, and then bound with anti-LdASS, anti-HGPRT and anti-TatD antibodies. (C) The crude organelle fraction of V5His-LdASS^WT^ over expressing amastigotes was further fractionated on a linear 20–70% sucrose gradient. Sucrose fractions 1–21 were assayed for the presence of LdASS by Western blot analysis using anti-V5 antibody. Here only fractions 1–14 are shown on the membrane, L: crude organelle fraction layered on the gradient, +: cleared lysate as positive control.

IFA experiments suggested that LdASS was found in a subcellular structure that was distinct from the microbody organelles containing the proteins HGPRT, LdIMPDH and LdPEX14 which define glycosomes [35]–[37]. In order to define the LdASS localization, we performed a subcellular fractionation of whole cell lysates of axenic *Leishmania* amastigotes from untransfected parasites or from parasites expressing V5His-LdASS^WT^. Both parasite strains showed a similar distribution of LdASS with a dual compartmentalization between cytosol (Fig. 6A, lane 2) and crude organelles (Fig. 6A, lane 3). For the later experiments, we used only the LdASS over expressing parasites since the distribution of over expressing LdASS mirrors the distribution of endogenous LdASS and the higher expression of recombinant ASS permits detection in further fractionation.

The degree of protection of ASS and various marker proteins from protease K digestion as an indication of sequestration in membrane-bound structures was evaluated in isolated crude organelles. The distribution pattern of LdASS, LdHGPRT (glycosomal marker) and LdTatD (cytosolic marker) is illustrated in Fig. 6B and shows that LdASS was present in both the cytosol (87%, quantified from Western blots normalized by the portion of each fraction loaded on the gel, see Materials and Methods) (Fig. 6B, lane 2) as well as in the crude organelles (13%, Fig. 6B, lanes 3–6). As expected, LdHGPRT and LdTatD were present exclusively in the crude organelles (Fig. 6B, lanes 3–5) and cytosol (Fig. 6B, lane 2) respectively. To verify that the proteins in the crude organelle fraction were protected in membrane bound structures, the crude organelles were treated with protease K. Interestingly, nearly 35% of LdASS present in the crude organelle fraction was protected from protease in the absence of detergent (4.6% of the total cellular ASS, Fig. 6B, lane 4) whereas in the presence of both protease and detergent, LdASS was completely degraded (Fig. 6B, lane 6). The protease sensitive LdASS present in the crude organelle fraction is approximately 8.4% of the total in the cell). The HGPRT found in the crude organelle fraction was protected from protease digestion as expected (Fig. 6B lane 4, middle panel). A higher enzyme concentration or a longer incubation time in the presence of PK did not reduce the amount of LdASS protected in the absence of detergent (data not shown). This result demonstrates that a fraction of LdASS is sequestered by a membrane protecting it from proteolysis.

In order to further define the structures containing ASS, crude organelles were fractionated over a linear sucrose gradient. Western blot analysis of the gradient fractions using anti-V5 antibodies showed that the V5His-LdASS was present in fractions 9–14 which correspond to a sucrose density of ∼38–60% (w/v) (Fig. 6C). Therefore, these studies reveal that LdASS, even though it has the putative glycosomal localization signal, is present mainly in the cytosol and a minor fraction is present in a glycosome-like vesicle.

### Parasites over expressing the inactive LdASS^G128S^ have reduced pathogenicity in mice

**Figure 7:**
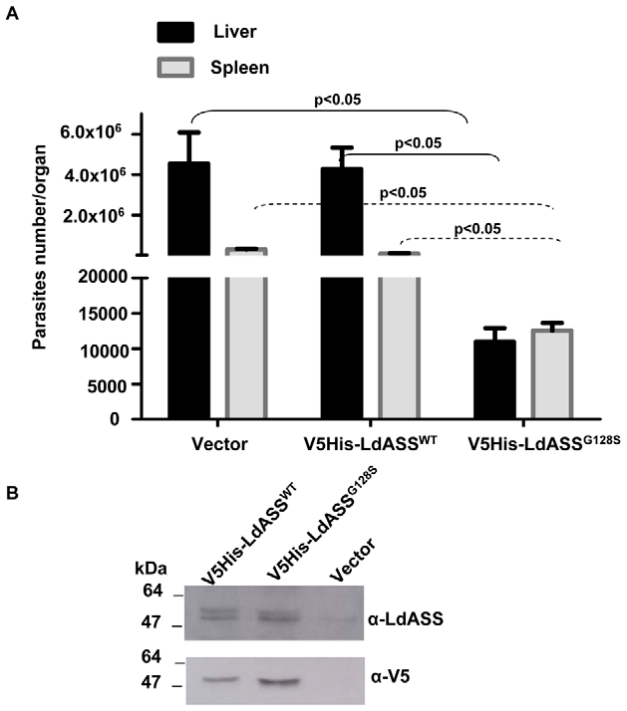
An inactive LdASS enzyme induces a loss of virulence *in vivo*. (A) Data presented are the average number of parasites per organ from 9 animals in liver (black bars) or spleen (gray bars). Error bars indicate the standard error. Means that are significantly different by One way ANOVA (Tukey HSD) are indicated. The experiment was repeated three times with similar results. (B) Western blot with anti-LdASS and anti-V5 antibodies using an equal number of lysed promastigotes (V5His-LdASS^WT^, V5His-LdASS^G128S^ and vector control) cultured for three weeks after recovery from 5 week infection of mice.

The higher expression of the LdASS enzyme in the amastigote stage suggested that ASS may be important for parasite survival and pathogenesis. To determine the role of LdASS in *Leishmania* pathogenesis, we infected susceptible BALB/c mice with either wild type parasites transfected with empty vector or parasites over expressing wild type or mutant LdASS gene. Five weeks after infection, the parasite load was determined from both liver and spleen by the serial dilution method. Vector control and V5His-LdASS^WT^ parasites survived well at 5 weeks post-infection in both liver and spleen (Fig. 7A). However, V5His-LdASS^G128S^ parasites had reduced survival in the animals showing significant reductions (p<0.05) of parasite burdens in both liver and spleen compared to the vector control parasite and LdASS^WT^ over expressing parasites. The altered virulence of the point mutant over expressing parasite can be attributed to the expression of the recombinant mutant LdASS^G1218S^ protein, which was confirmed by Western blot analysis in the absence of drug selection *in vitro* after recovery of parasites from mice (Fig. 7B). The mouse infection experiment was repeated twice with similar findings. We demonstrate here that dominant negative effect of over expression of the non-functional LdASS^G128S^ leads to a reduction of pathogenicity in mice (400 and 8 fold less, in liver and spleen respectively compared to LdASS^WT^ transfected; 413 and 23 times less, in liver and spleen respectively compared to the vector control transfected). These data suggest that LdASS is essential for parasite survival and growth in the mammalian host.

## Discussion

In previous studies aimed at identifying biomarkers associated with the attenuation of the *centrin* gene-deleted cell line, we found that the argininosuccinate synthase (ASS) was down regulated in the amastigote stage when cell division is disrupted in this cell line [2], [4]. Furthermore, expression studies at the RNA or protein level demonstrated a higher level of ASS in the life cycle stage responsible for the disease in wild type parasites [4], thus ASS may constitute a suitable target for anti-leishmaniasis chemotherapy or vaccine design. In this study, we report a detailed characterization of ASS in *Leishmania donovani*. ASS has been described in several organisms ranging from archaebacteria to higher eukaryotes suggesting a common ancestor [10], [43]–[44]. Although fully described in those organisms, no studies on *Leishmania* ASS have been reported. The predicted *L. donovani* ASS sequence shows a significant level of similarity in primary amino-acid sequence with its mammalian homolog (59.7%) and displays the same functional domains. The presence of a nucleotide-binding domain, the synthetase domain, and a C-terminal oligomerization domain [13] supports this homology. The observation that ASS is only found in *Leishmania* among the *Trypanosomatidae* may be explained by the acquisition of this gene in *Leishmania* by lateral transfer from bacteria [9] or its loss in the common ancestor of trypanosomes after their divergence from *Leishmania* as suggested for other genes i.e. *Leishmania* Phenylalanine hydroxylase (PAH) [45]. Due to the presence of ASS in *Leishmania* only, but not in the Trypanosomes, we hypothesize that *Leishmania* kept this gene during evolution because of its importance for parasite survival.

In our study, we showed that LdASS has argininosuccinate synthase activity *in vitro* and a single point mutation in the ATP binding domain is sufficient to abolish this activity. The specificity of enzyme activity was confirmed by HPLC identification of argininosuccinic acid (ASA) in the reaction products catalyzed by V5His-LdASS^WT^ but not by the point mutant. Similar analyses of point mutations in human ASS showed that *in vitro* enzyme activity of recombinant proteins correlated with severity of disease in patients carrying those mutations [46]–[47]. The correlation between severe disease and lack of enzyme activity shown by Berning [47] suggests that the severe, sometimes fatal disease associated with G117S mutation implies an inactive ASS, though no functional studies were reported on that particular mutation [25]. Our *in vitro* activity assay demonstrates the loss of function when the corresponding G is replaced with an S in the *Leishmania* enzyme. The specificity of activity of LdASS was supported further by blocking with α-methyl-DL-aspartic acid (MDLA), an ASS specific inhibitor [42], [48]–[49]. MDLA has been shown to be a specific and effective inhibitor of ASS from a variety of organisms [31], [50] and in our study of *Leishmania* ASS activity. Taken together, the protein encoded by the gene that we identified is a genuine ASS in *Leishmania*. Our observation that MDLA does not inhibit growth in culture even though enzyme activity is blocked *in vitro* may be explained by failure of the drug to penetrate to the site of localization of LdASS. Pursuing LdASS as a drug target or vaccine candidate will benefit from knowing more about its localization and function in *Leishmania*.

Many of the biochemical pathways in *Leishmania* are compartmentalized into subcellular structures that contain the relevant components for example: glycosomes [19], [51]; mitochondria [52]–[54] and acidocalcisomes [55]–[56]. The tripeptide at ASS's carboxy terminal, SSL, a variant of the PTS1 signal [57]–[58], has been described as a targeting signal to glycosomes [16]. Protein targeting to the peroxisomal/glycosomal matrix includes the recognition of PTS1 by a specific receptor PEX5, followed by interaction with the docking protein PEX14 [38], [59]. Our *in vitro* binding assay experiments show the presence of a free SSL at the carboxy terminal end of LdASS is required for its binding to PEX5. Subcellular fractionation studies show that ASS appears to have a dual compartmentalization. LdASS seems to be localized partly in a vesicle as suggested by a glycosomal targeting signal, its protection from proteolysis by Protease K (4.6% of total cell quantity) and its presence in the high percentage fractions of a sucrose gradient. However, the majority of LdASS is cytosolic (87%). The punctate distribution in IFA may include a portion of LdASS in membrane bound vesicles. The remainder of the ASS that is cytosolic may be so diffusely distributed at low concentration that it is not readily visible in immunofluorescent images. Alternatively, some of the cytosolic portion may be present in clusters too large to be resolved on a 4% native gel that fractionate with the crude organelles, but are susceptible to protease digestion (8.4% of total cellular ASS).

The vesicles containing a small percentage of total LdASS are not the classical glycosomes because of the lack of co-localization of LdASS with glycosomal marker proteins such as HGPRT, PEX14 or IMPDH as indicated by IFA images. Growing evidence is suggesting that the presence of a consensus tripeptide PTS1 signal does not guarantee import through the peroximal membrane [60]–[62]. Moreover even if a given PTS1 harboring protein is able to interact with PEX5 *in vivo*, the PEX5-PTS1 cargo protein complex will sometimes not be imported into peroxisomes [63]. In contrast, some proteins can be localized to perixosomes even if their original sorting signals are masked [63]. Thus LdASS may be among those proteins that have a PTS1 signal that interacts with PEX5, as we demonstrated, but is not imported into a vesicle efficiently.

The LdASS-containing vesicle is likely to have new physical properties that need further investigation. Thus if the PEX5 binding of LdASS is the first step to a vesicular import, a novel PEX14-like protein may be found on a novel vesicle. The fact that known glycosomal proteins cosediment at ∼55% sucrose [23] and the spread of LdASS in fractions with 38–60% sucrose, suggests that ASS is within a novel vesicle that shares physical properties with glycosomes. Identification of the novel vesicle and/or the cytosolic cluster and its components in addition to ASS is the subject of future studies to understand the function of ASS and its role in *Leishmania* pathogenesis.

ASS is a key enzyme of the urea cycle and the absence from the *Leishmania* genome of two key enzymes in the urea cycle, ornithine carbomyl transferase and argininosuccinate lyase, suggests that no true Ureotelism (complete urea cycle) seems to occur in this species [64] despite the demonstration of urea excretion in *L. braziliensis* [65]. The culture of *L. donovani* in serum free media lacking 5 amino-acids, in pathways involved in arginine biosynthesis (Arg, Ala, Asp, Glu and Gln) showed that aspartate and citrulline could not substitute for arginine (Fig. S2).

L-Arg is an essential amino acid for *Leishmania* growth and *Leishmania* promastigotes cannot be maintained in L-Arg-free media [66]. The absence of growth of *L. donovani* in medium supplemented with aspartate and citrulline but lacking arginine confirms the absence of enzymes capable of effecting the conversion of argininosuccinate into arginine by alternate pathways and suggests that argininosuccinate product serves another purpose in this organism.

Since LdASS is expressed in amastigotes at higher levels compared to promastigotes, we speculated that LdASS has a role in *Leishmania* pathogenesis as has been the case with many of the amastigote specific genes (A2, CPB, Ldp27) [28], [67]–[69] Therefore, deletion of ASS alleles by homologous recombination would be desirable to further characterize the function of ASS in *Leishmania*. Our attempts to make ASS deleted parasites have been unsuccessful to date. Clones recovered after the second allele knock-out had amplified copies of the integrated drug resistance marker suggesting instability of this genomic region. Furthermore, these clones retained a native chromosomal copy of the ASS gene (data not shown). Deep sequencing results indicate that *L. donovani* (*Ld1S2D*) is triploid on Chromosome 23 (personal communication, Dr. Peter Myler and [70]). Creating ASS deleted parasites to understand the role of LdASS in *Leishmania* pathogenesis is the goal of future studies.

Alternatively to test the potential of LdASS as a virulence factor, we explored the potential of the dominant negative effect of over expressing the mutant form of LdASS, which lacks enzymatic activity. This approach has been successful in exploring the functional role of other *Leishmania* proteins [3], [71]. Over expression of the non functional LdASS^G128S^ altered the severity of the disease as demonstrated by a significant reduction of parasite load in spleen and liver. The genetically altered parasites retain ASS-expressing episomes throughout the course of infection since the recovered parasites still express V5His-LdASS. The greater abundance of the non-functional ASS in a parasite cell that has the endogenous functional ASS can have a dominant negative effect because ASS is known to be active as a tetramer [13]. Also, the mutant enzyme is likely to be targeted to the correct site of the endogenous LdASS because the mutant LdASS has the similar distribution as the endogenous ASS in IFA and it binds equally well to the PEX5 receptor in the import pathway. Thus our data suggest that the biological role of *Leishmania* ASS promotes amastigote survival in the host and may be required for pathogenicity.

LdASS may also be proposed as a target for drug design. This will require approaches that target LdASS while minimally toxic to the homologous human ASS. Approaches that could disrupt the unique localization of LdASS and the unusual pathway in which it is involved or small molecule screening that could identify an inhibitor with higher binding to the *Leishmania* enzyme may prove effective.

In conclusion, we identified here an argininosuccinate synthase enzyme in *Leishmania* that was demonstrated to play a role in parasite virulence. Although highly similar to its human homolog, the divergence of the metabolic pathways between human and *Leishmania*, the availability of specific inhibitors and the reduction of pathogenesis of a parasite expressing an inactive protein, make ASS an attractive target of therapeutic drugs for leishmaniasis treatment.

## Supporting Information

Table S1 Primers used in the study.
(DOC)

Figure S1 Sequence alignment of Argininosuccinate Synthase with human (hASS) and *Leishmania donovani* (LdASS). The predicted amino acid sequence of LdASS was aligned with its human homolog hASS (NP_000041) using the ESPript program [72]. The nucleotide binding domains, the synthetase domain and the glycosomal targeting signal are underlined in blue, gray and red, respectively. Residues involved in substrate binding are marked with a black star. The G117 in human sequence is conserved with its homolog in *Leishmania* (G128) and marked with a red star.
(TIF)

Figure S2 *In vitro* growth kinetics of *L. donovani* promastigotes. *Ld1S2D* parasites were grown in the chemically defined media [73] in different conditions: depleted: M199 lacking 5 amino acids (Arg, Ala, Asp, Glu and Gln); 5 AA added: M199 reconstituted with those 5AA; Asp/cit: depleted media supplemented with Aspartate and citrulline, Arg: depleted media supplemented only with Arginine; Asp: depleted media supplemented with Aspartate; FBS: complete medium with fetal bovine serum (10%). Population densities were calculated from triplicate cultures represented by different colors on line graphs. The values represents mean +/− SD. Adapted parasites were inoculated to a final concentration of 1×l0^6^ cells/mL in 5 mL fresh medium in 25 cm^2^ plastic tissue culture flasks, in triplicates. Twenty-microliter aliquots were taken daily and diluted in Isoton II and parasite density was determined using a Coulter Counter.
(TIF)
