# Supplementary material for: Leishmania donovani Argininosuccinate Synthase Is an Active Enzyme Associated with Parasite Pathogenesis
Source: PLoS Negl Trop Dis. 2012 Oct 18;6(10):e1849. doi: 10.1371/journal.pntd.0001849 (PMC3475689; doi:10.1371/journal.pntd.0001849)
Supplement: Table S1 — Primers used in the study. (DOC) [file pntd.0001849.s001.doc]

**Table 1**: Primers used in the study.

| **Primer Name** | **Sequence** | **Use** |
| --- | --- | --- |
| **F1** | 5’-ATGCCTGCAACGGCGACGGAG-3’ | Forward primer for cloning of ASS^WT^ and ASS^G128S^ in pCRT7-CT. |
| **R1** | 5’-CAAGCTGCTCGGCATCTCCTTCG-3’ | Reverse primer for Cloning of ASS^WT^ and ASS^G128S^ in pCRT7-CT |
| **F2** | 5′-ATCTGCCACAGCGCCACGGGGAAG-3’ | Forward primer to introduce the G128>S mutation |
| **R2** | 5’-CCCCGTGGCGCTGTGGCAGATCGC-3’ | Reverse primer to introduce the G128>S mutation |
| **F3** | 5’-AAGGGCAATTCGAAGCTTGAAGGTAAGCCTA  TCCCTAACCCTCTCCTCGGTCTCGATTCTACGCGTACCGGTCCTGCAACGGCGACGGAGGTT-3’ | Forward primer for cloning of ASS^WT^ in pEXP-5-NT.  Contains V5 epitope |
| **R3** | 5’- TCACAAGCTGCTCGGCATCTCCTT-3’ | Reverse primer for Cloning of ASS^WT^ in pEXP-5-NT |
| **F4** | 5’- TGACTAGTATGTCTGGTTCTCATCATCAT-3’ | Forward primer for cloning NH_2_ constructs into pkSNeo |
| **R4** | 5’-TGACTAGTTCACAAGCTGCTCGGCATCTC-3’ | Reverse primer for cloning NH_2_ constructs into pkSNeo |
| **F5** | 5’-TCAACTAGTATGCCTGCAACGGCGACGGAG-3’ | Forward primer for cloning COOH constructs into pkSNeo |
| **R5** | 5’-AGTACTAGTTTAACCGGTACGCGTAGAATCG  AG-3’ | Reverse primer for cloning COOH constructs into pkSNeo |
