# Supplementary figures and images for: Leishmania donovani Argininosuccinate Synthase Is an Active Enzyme Associated with Parasite Pathogenesis
Source: PLoS Negl Trop Dis. 2012 Oct 18;6(10):e1849. doi: 10.1371/journal.pntd.0001849 (PMC3475689; doi:10.1371/journal.pntd.0001849)

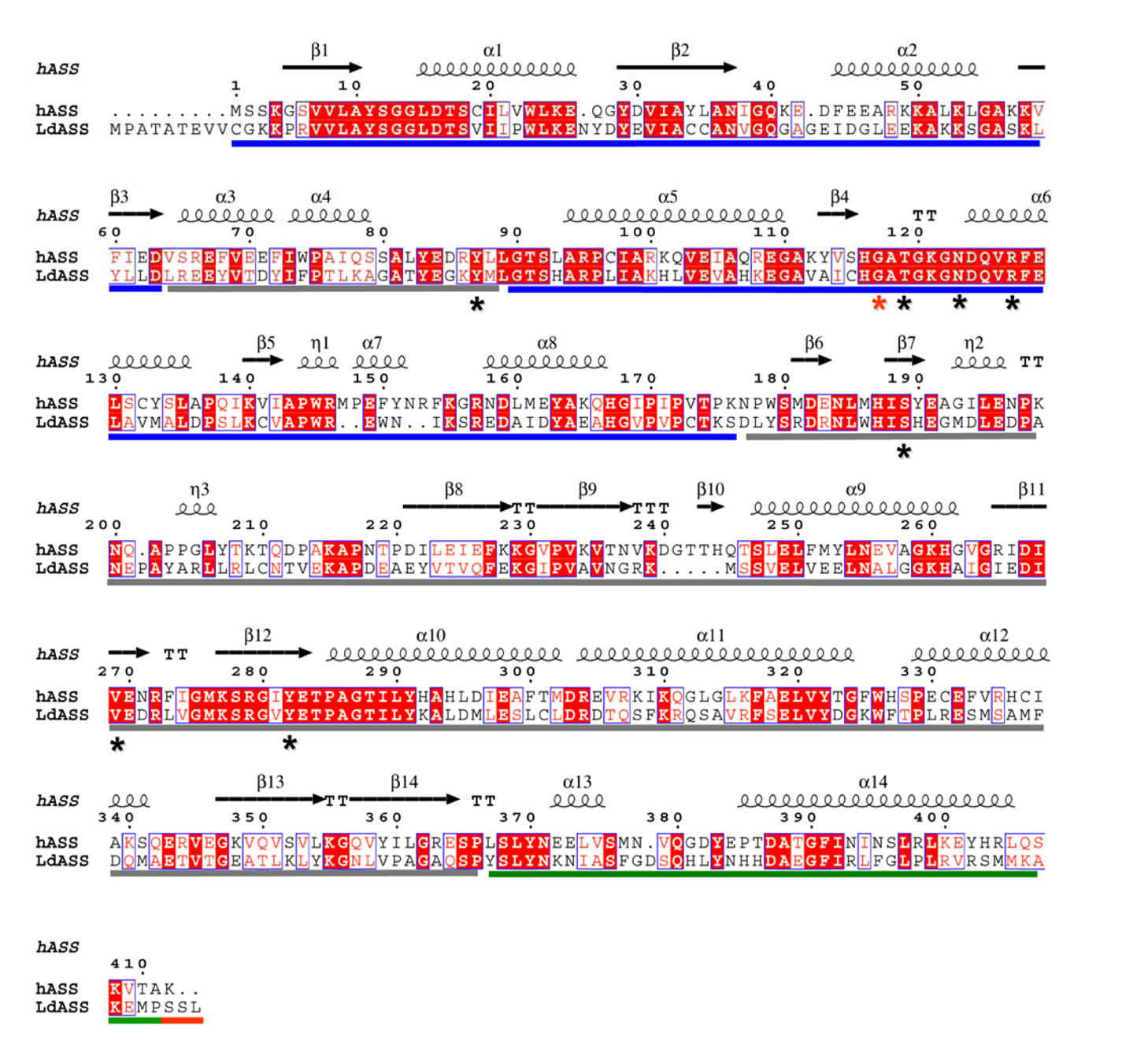

Supplement: Figure S1 — Sequence alignment of Argininosuccinate Synthase with human (hASS) and Leishmania donovani (LdASS). The predicted amino acid sequence of LdASS was aligned with its human homolog hASS (NP_000041) using the ESPript program [72]. The nucleotide binding domains, the synthetase domain and the glycosomal targeting signal are underlined in blue, gray and red, respectively. Residues involved in substrate binding are marked with a black star. The G117 in human sequence is conserved with its homolog in Leishmania (G128) and marked with a red star. (TIF) [file pntd.0001849.s002.tif]

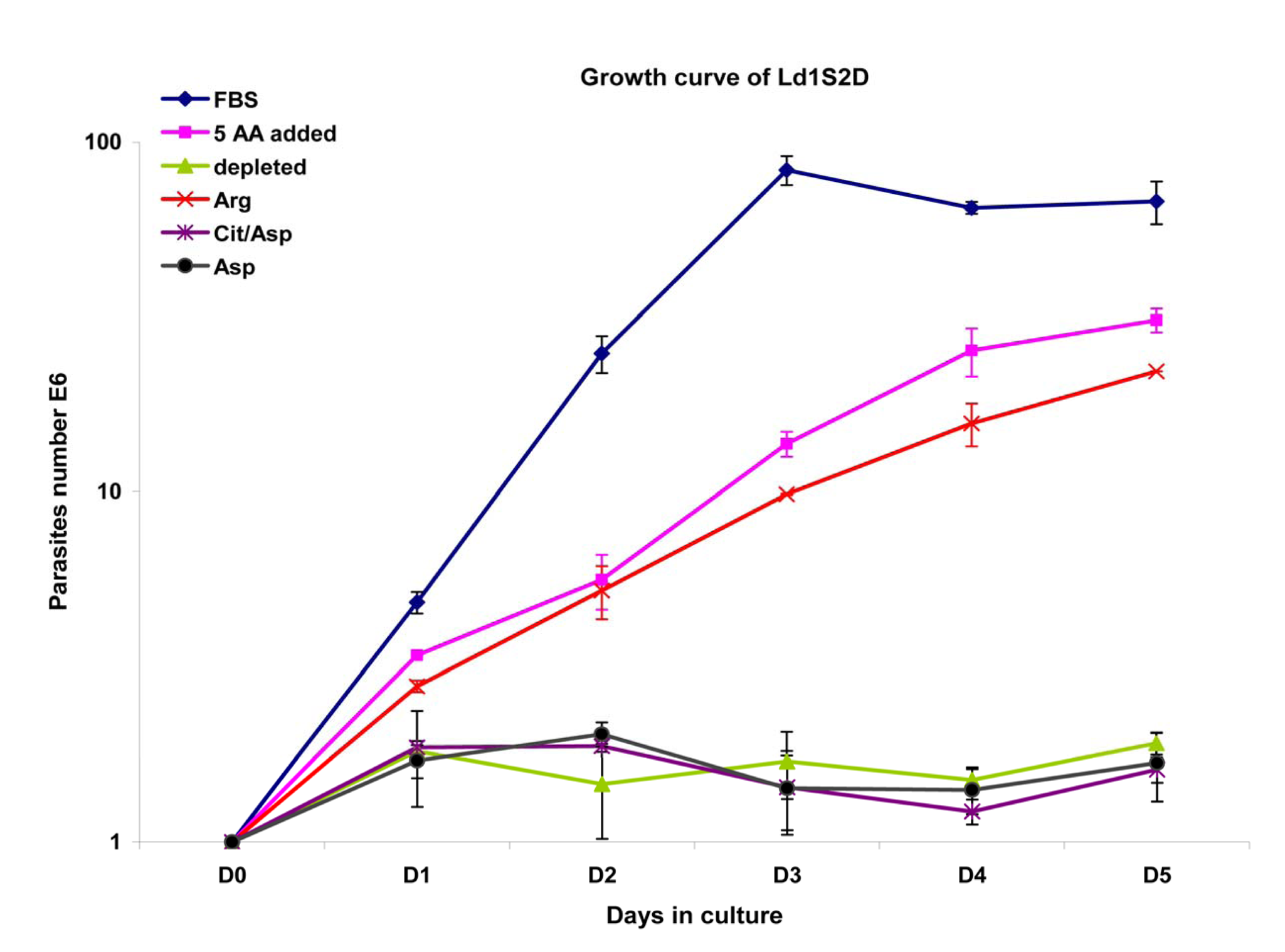

Supplement: Figure S2 — In vitro growth kinetics of L. donovani promastigotes. Ld1S2D parasites were grown in the chemically defined media [73] in different conditions: depleted: M199 lacking 5 amino acids (Arg, Ala, Asp, Glu and Gln); 5 AA added: M199 reconstituted with those 5AA; Asp/cit: depleted media supplemented with Aspartate and citrulline, Arg: depleted media supplemented only with Arginine; Asp: depleted media supplemented with Aspartate; FBS: complete medium with fetal bovine serum (10%). Population densities were calculated from triplicate cultures represented by different colors on line graphs. The values represents mean +/− SD. Adapted parasites were inoculated to a final concentration of 1×l06 cells/mL in 5 mL fresh medium in 25 cm2 plastic tissue culture flasks, in triplicates. Twenty-microliter aliquots were taken daily and diluted in Isoton II and parasite density was determined using a Coulter Counter. (TIF) [file pntd.0001849.s003.tif]
